# Combating resistance to DNA damaging agents

**DOI:** 10.18632/oncoscience.423

**Published:** 2018-06-24

**Authors:** Jung-Suk Choi, Anthony Berdis

**Affiliations:** Department of Chemistry, Cleveland State University, Cleveland, OH 44115, USA; Center for Gene Regulation in Health and Disease, Cleveland State University, Cleveland, OH 44115, USA

**Keywords:** chemotherapy, cancer, mutagenesis, polymerases, glioblastoma

**Figure 1:**
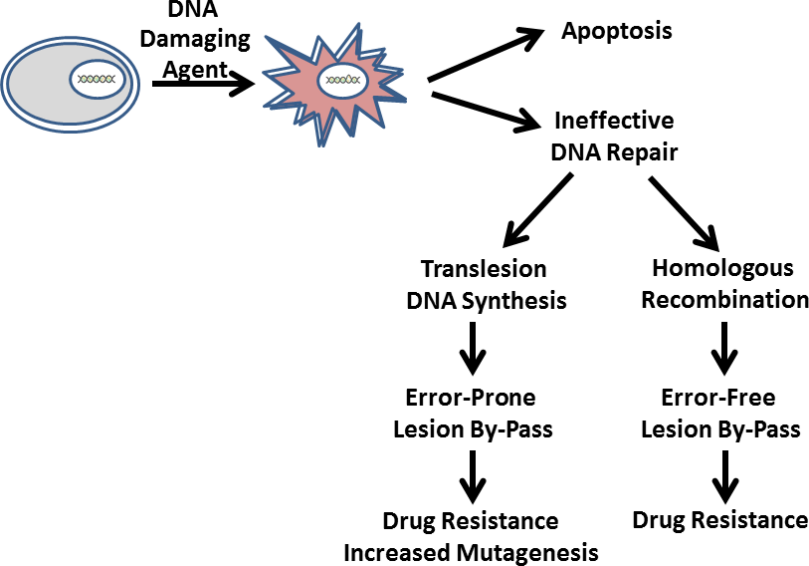
In the absence of effective DNA repair, cancer cells can by-pass unrepaired DNA lesions via homologous recombination or by translesion DNA synthesis While both pathways can generate clinical resistance to DNA damaging agents, translesion DNA synthesis is more deleterious as this an error-prone process can drive mutagenesis, genetic drift, and tumor recurrence.

DNA damaging agents are widely used in oncology to treat both hematological and solid cancers. Some commonly used modalities include ionizing radiation, platinum drugs (cisplatin, oxaliplatin, and carboplatin), cyclophosphamide, chlorambucil, and temozolomide. By modifying the chemical structure of nucleic acid, these agents induce apoptosis to subsequently eliminate cancer cells from the body. Unfortunately, the efficacy of these agents can be significantly reduced by various factors that drive drug resistance. For example, increases in drug efflux and/or increased drug metabolism can lower the intracellular concentration of an anti-cancer agent thereby reducing its ability to inflict enough DNA damage to induce apoptosis. Another mechanism involves deletions or mutations in proteins associated with several DNA repair pathways that respond to damaged DNA. For example, cancers such as Li-Fraumeni Syndrome and Lynch Syndrome (formerly referred to as hereditary non-polyposis colorectal cancer (HNPCC)) possess mutations in p53, a key regulator in DNA damage response or DNA mismatch repair, respectively [1, 2]. In these cases, the inability of a cancer cell to appropriately respond to DNA damage or repair it allows an oncogenic cell to survive the cellular insults caused by DNA damaging agents. Moreover, cancer cells that survive these insults are more likely to undergo cell division and proliferate rather than die via apoptosis. This occurs as unrepaired DNA lesions are effectively by-passed by two mutually exclusive pathways (Figure 1). The first involves homologous recombination which, in most cases, allows for “error-free” by-pass of a lesion. The alternative pathway reflects the ability of DNA polymerases to efficiently insert nucleotides opposite and beyond a DNA lesion. This activity, termed translesion DNA synthesis (TLS), can be highly pro-mutagenic and generate more mutations in a cancer cell [3]. In turn, higher mutation frequencies can create more aggressive cancers and/or lead to tumor recurrence. An unfortunate example of this phenomenon occurs during the treatment of patients diagnosed with glioblastoma multiforme (GBM). Standard treatments for GBM include administration of the DNA alkylating agent, temozolomide. While this drug is initially effective in reducing tumor burden, its efficacy typically diminishes within a year due to the emergence of drug resistance caused by mutagenesis of proteins such as those involved in DNA repair [4]. Indeed, a recent report highlights a role for TLS activity in generating resistance as temozolomide-treated tumors display higher mutation rates (∼90 mutations/Mb) compared to initial untreated tumors (<4 mutations/Mb) [5]. Furthermore, these hypermutation rates coincided with mutations in key genes associated with DNA mismatch repair, retinoblastoma, and mammalian target of rapamycin (mTOR) [5].

In a series of recent publications, we evaluated whether inhibiting TLS activity could improve the efficacy of DNA damaging agents such as temozolomide [6, 7]. The approach was to use a unique artificial nucleoside analog designated 5-nitroindoyl-2′-deoxyriboside (5-NIdR) to terminate the replication of DNA lesions such as abasic sites which are produced by temozolomide. Applying this strategy in cell-based models of leukemia and brain cancer, we demonstrated that combining 5-NIdR with a sub-lethal dose of temozolomide produces synergistic increases in apoptosis compared to treatment with either temozolomide or 5-NIdR used individually. In addition, GBM cells treated with temozolomide and 5-NIdR display higher levels of pATM and pH2AX compared to cells treated with either agent alone. These increases coincide with a cell-cycle block at S-phase, suggesting that 5-NIdR inhibits the inappropriate replication of damaged DNA. This conclusion was validated using a fluorogenic derivative of 5-NIdR that detected incorporation of the nucleotide analog into genomic DNA only after temozolomide treatment. Furthermore, *in vitro* nucleotide incorporation assays demonstrated that several high- and low-fidelity human DNA polymerases efficiently utilize the triphosphate form of 5-NIdR when replicating an abasic site. Collectively, these biochemical and cell-based studies provide evidence for the ability of the artificial nucleoside to inhibit the replication of DNA lesions produced by temozolomide.

Equally important, our pre-clinical studies using a xenograft mouse model demonstrated that 5-NIdR can function as an adjunctive therapeutic agent with temozolomide to treat GBM. Results showed that tumor-bearing mice treated with temozolomide displayed a ∼4-fold delay in tumor growth compared to vehicle-treated animal. However, combining 5-NIdR with temozolomide caused complete tumor regression in >65% of mice while producing a delay in tumor growth in the remaining 35%. In addition, preliminary toxicology studies showed that repeat dosing with high doses of 5-NIdR did not produce adverse effects on hematological or major organ function. Current investigations focus on pre-clinical studies using intracranial brain cancer models to confirm that 5-NIdR effectively crosses the blood brain barrier and potentiates the efficacy of temozolomide. These efforts are especially important as approximately 4,000 children and 20,000 adults in the United States are diagnosed with a brain tumor each year. Developing compounds that increase the efficacy of temozolomide may improve treatment of this disease. Clearly this is important as brain cancers are the deadliest of all cancers, having 5-year survival rates of less than 5% and a median survival time of less than 16 months [8].

Another important question is whether or not this approach can be extended to DNA damaging agents other than temozolomide to provide better treatments against other cancers? A relevant is ionizing radiation therapy which is used in ∼50% of all cancer patients. The primary advantage of ionizing radiation lies in the ability to attack tumors that are typically inaccessible to surgical removal. This is achieved by precisely focusing the ionizing beams at the tumor which also minimizes injury to surrounding healthy tissue. At the cellular level, ionizing radiation primarily generates double strand DNA breaks, a non-instructional DNA lesion that is similar to an abasic site. Double strand DNA breaks are processed by different repair pathways that require the activity of several DNA polymerases. However the polymerases involved in processing double strand DNA breaks are distinct from those involved in replicating abasic sites. Regardless, it will be of significant interest to evaluate if artificial nucleosides such as 5-NIdR can inhibit these polymerases to potentiate the cell-killing effects of ionizing radiation.
